# Targeting ADAR stability: a novel approach to combat glioblastoma

**DOI:** 10.3389/fonc.2026.1827793

**Published:** 2026-08-06

**Authors:** Qin Fei, Jueru Huang, Yufeng Zhang, Yi He, Qiang Fu

**Affiliations:** Department of Anesthesiology, The Third People’s Hospital of Chengdu, Chengdu, Sichuan, China

**Keywords:** ADAR, cell migration, glioblastoma, RNA Editing, STUB1, ubiquitination

## Abstract

**Background:**

Adenine deaminase (ADAR) is a key RNA editing enzyme that plays an important role in the initiation and progression of cancer. However, the mechanisms regulating its protein stability and its function in glioblastoma (GBM) remain unclear.

**Methods:**

This study systematically investigated the expression patterns of ADAR across pan-cancer types and its specific mechanisms in GBM through the integration of multi-omics data analysis and experimental validation. We performed bioinformatics analysis on the TCGA dataset, utilising single-cell RNA sequencing data, *in vitro* functional experiments, and molecular studies including co-immunoprecipitation and ubiquitination detection.

**Results:**

We found that ADAR exhibits significant differential expression across various cancers and is closely associated with tumour mutation burden and survival outcomes. Further analysis revealed that ADAR is highly expressed in GBM and primarily enriched in tumour cells, with its expression levels significantly positively correlated with key cancer signalling pathways such as PI3K-AKT-mTOR and G2M checkpoints. Functional experiments confirmed that ADAR significantly promotes the migratory capacity of glioblastoma cells. Importantly, we identified E3 ubiquitin ligase STUB1 as a specific regulator of ADAR, which mediates ADAR’s proteasome-dependent degradation by promoting K48-linked ubiquitination of ADAR. Knocking down STUB1 significantly upregulates ADAR protein levels and enhances tumour cell migration capacity.

**Conclusions:**

This newly identified STUB1-ADAR regulatory axis not only elucidates the molecular mechanisms underlying ADAR homeostasis but also provides new potential strategies for targeted therapy against glioblastoma.

## 1 Introduction

Adenosine deaminase ADAR family members, as key regulatory factors in RNA editing, have garnered significant attention in recent years for their complex roles in tumour biology (1, 2). While previous studies have explored the functions of ADAR1 in pan-cancer analyses and the immune microenvironment from different perspectives (3, 4), little is known about the specific functional mechanisms of ADAR1 protein in post-transcriptional regulatory networks in tumours, particularly its stability regulation and precise mechanisms in cell migration processes.

Glioblastoma (GBM) is the most common malignant tumour type in the central nervous system, with a poor prognosis and a median survival of only 15 months (5, 6). Although previous studies have shown that ADAR1 is abnormally expressed in certain gliomas (7, 8), how ADAR1 maintains its expression levels in GBM through protein stability regulation mechanisms, and how this regulation influences tumour cell migration capacity at the molecular level, remains an unresolved scientific question.

The ubiquitin-proteasome system (UPS) serves as the primary protein degradation pathway in cells and plays a crucial role in tumourigenesis (9–11). STUB1 (CHIP), a molecular chaperone protein with E3 ubiquitin ligase activity, promotes the degradation of substrate proteins by catalysing their ubiquitination (12, 13). Interestingly, our preliminary studies revealed an interaction between STUB1 and ADAR1, suggesting that STUB1 may participate in regulating ADAR1 protein stability and thereby influence its functional activity.

This study aims to elucidate the mechanism of a novel STUB1-ADAR1 regulatory axis in GBM. Unlike previous studies that focused on the transcriptional expression patterns and immune-related functions of ADAR1, we focus on the post-translational modification level to explore how STUB1 precisely regulates ADAR1 protein stability through the ubiquitin-proteasome pathway and the impact of this regulatory mechanism on GBM cell migration ability.

Based on our preliminary findings from single-cell sequencing data analysis and *in vitro* experiments, ADAR1 exhibits significant expression differences across different GBM samples, and its expression levels are closely associated with tumour cell migration capacity. More importantly, we discovered that STUB1 can directly interact with ADAR1, promoting its ubiquitination modification and proteasome-dependent degradation, thereby inhibiting ADAR1-mediated RNA editing activity and its function in promoting tumour cell migration.

## 2 Materials and methods

### 2.1 Reagents

All pharmacological reagents used in this study were dissolved in dimethyl sulfoxide (DMSO), dispensed and stored at -80 °C until use. Cells were treated with the following inhibitors 6 h before collection: 3-methyladenosine (3MA, autophagy inhibitor, HY-19312, MCE, USA) at a final concentration of 1 μM; chloroquine (CQ, autophagy inhibitor, HY-17589A, MCE, USA) at a concentration of 20 μM; and Z-VAD-FMK (Z-VAD, pan-caspase inhibitor, T7020,Topscience Co. Ltd, China) at a concentration of 10 μM; and MG132 (proteasome inhibitor, HY-13259, MCE, USA) at a concentration of 10 μM.

### 2.2 ADAR transcript abundance evaluation across multiple datasets

The expression levels of the ADAR gene in various human cancers from The Cancer Genome Atlas (TCGA) are obtained from the UALCAN database (https://ualcan.path.uab.edu/index.html). Furthermore, the mRNA expression levels of the ADAR gene in various non-matched tumours and corresponding normal tissues were analysed using the Sangerbox data analysis platform (http://sangerbox.com/). The expression levels of ADAR in central nervous system tumours were analysed using the Kaplan-Meier Plotter (https://kmplot.com/). This analysis was conducted using human cancer samples and matched normal tissues. Cancer type abbreviations from TCGA are provided in **Supplementary Table 1**.

### 2.3 Prognostic analysis of ADAR

The Kaplan-Meier plotter database was utilised to conduct a Kaplan-Meier survival analysis. Patients were categorised as high or low expressors based on the median value of ADAR gene expression levels.

### 2.4 Protein expression analysis of ADAR in glioblastoma

To determine the protein expression levels of ADAR in glioblastoma, immunohistochemical (IHC) images were obtained from the https://www.proteinatlas.org/ (THPA) database.

### 2.5 Gene mutation and ADAR expression

We retrieved standardized pan-cancer data from the UCSC Xena platform (https://xenabrowser.net/), specifically the TCGA Pan-Cancer dataset (PANCAN, N = 10535, G = 60499), from which we extracted expression data for ADAR (ENSG00000160710) across all samples. We restricted our analysis to specimens classified as “Primary Blood Derived Cancer-Peripheral Blood” and “Primary Tumour,” and additionally acquired Level 4 Simple Nucleotide Variation data processed by MuTect2 software (14) for all TCGA samples from the Genomic Data Commons portal (https://portal.gdc.cancer.gov/). After integrating mutation and gene expression data, we filtered out synonymous mutations, applied log2(x+0.001) transformation to expression values, and excluded cancer types with fewer than three samples, ultimately yielding expression data for 15 distinct cancer types. Statistical analyses were conducted using R software (version 4.0.3), employing non-paired Wilcoxon Rank Sum and Signed Rank Tests for pairwise differential expression analysis between clinical stages, and the Kruskal-Wallis test for multi-group comparisons. For the analysis of ADAR expression in relation to the mutation landscape in GBM multiforme, we included 153 mutation-profiled samples (with 128 samples [83.7%] represented in the visualization) and applied chi-square tests to evaluate differences in gene mutation frequencies between expression-defined groups.

### 2.6 Pan-cancer analysis of the relationship between the ADAR gene expression and tumour heterogeneity

The data set under investigation comprised STAR-counts data and corresponding clinical information for 33 tumours, which were downloaded from the TCGA database (https://portal.gdc.cancer.gov). Subsequently, the data was converted to TPM format and standardised using a log2 (TPM + 1) transformation. Following the selection of samples containing both RNA sequencing data and clinical information, further analysis was performed. TMB is derived from the article “The Immune Landscape of Cancer”, which was published by Vesteinn Thorsson et al. in 2018 (15). The abbreviation MSI is derived from the article “Landscape of Microsatellite Instability Across 39 Cancer Types”, which was published by Russell Bonneville et al. in 2017 (16). The statistical analysis was conducted using R software, version 4.0.3. Statistically significant results were defined as those with a p-value less than 0.05.

### 2.7 Correlation between ADAR expression and pathways

The analysis was performed using the “ssgsea” parameter in the GSVA R package. The ssGSEA algorithm was utilised to calculate the enrichment scores for each pathway in each sample, thereby establishing the correlation between the samples and the pathways. By calculating the correlation between gene expression and pathway scores, it is possible to determine the relationship between ADAR and the relevant pathway.

### 2.8 Pathway analysis

Enrichment analysis for GO and KEGG were performed with the GeneCodis tool (https://genecodis.genyo.es/). ADAR-associated genes identified in glioblastoma were utilized as input for enrichment analysis. The genes were extracted from the UALCAN database and subjected to enrichment analysis, with the top 10 significantly enriched functional categories presented.

### 2.9 Protein-protein docking

In this study, molecular docking method was used to predict the interaction between ADAR and STUB1 proteins. Firstly, the amino acid sequences of the target proteins were obtained by searching in the UniProtKB database based on the gene names. Subsequently, the obtained sequences were inputted into the SWISS-MODEL online server for homology modelling to construct a 3D structural model of the target protein, and the construction results were saved in a PDB format file.ADAR-STUB1 docking analyses were executed using GRAMM software. The software performs rigid docking based on the principle of geometric complementarity, i.e., the protein structures of the ligand and receptor are kept free of conformational changes during the docking process, and only potential complementary binding sites are searched on the protein surface. After the docking calculation is completed, the system generates a total of 10 possible docking conformations. The results showed that the first-ranked docking conformation exhibited the optimal binding affinity with a binding energy of -10.6 kcal/mol, suggesting that this conformation may represent the most favourable interaction mode between ADAR-STUB1.

### 2.10 Cell lines and culture

The cell lines used in this study included NHA, T98G, LN18, U251, LN229, U87 and HEK 293T, which were cultured in DMEM (Basal Media, Shanghai, China). The media were supplemented with 10% foetal bovine serum (FBS) (Gibco, Carlsbad, CA). All cells were cultivated at a temperature of 37 degrees Celsius within a humidified incubator containing 5% CO2.

### 2.11 Inhibitor treatments

To investigate the protein degradation pathways, cells were treated with the following inhibitors. Three pathway inhibitors were used: the pan-caspase inhibitor Z-VAD (10 μM, TargetMol), the ubiquitin-proteasome pathway inhibitor MG132 (20 μM, MCE), and the autophagy inhibitors 3-methyladenine (3-MA, 1 mM, MCE) or chloroquine (CQ, 20 μM, Sigma-Aldrich). Cells were treated with the above inhibitors for 6 h. In all inhibitor treatment experiments, an equal volume of dimethyl sulfoxide (DMSO) was used as the vehicle control. For co-immunoprecipitation (Co-IP) experiments, cells were treated with MG132 (20 μM, MCE) for 4 h before harvest.

### 2.12 RNA extraction and quantitative real‐time PCR analysis

The RNA was extracted using a total RNA extraction reagent (Vazyme Biotech). The reverse transcription process was carried out utilising a GoScript Reverse Transcription System (Promega, Madison, WI, USA). Real-time quantitative polymerase chain reaction (qPCR) was performed with qPCR Master Mix (Vazyme Biotech) in an Applied Biosystems 7500 Real-Time PCR System. The primers employed in this study were designed by Sangon Biotech (Shanghai, China), ADAR, forward: 5’-TCCGTCTCCTGTCCAAAGAAGG-3’ and reverse: 5’-TTCTTGCTGGGAGCACTC.

ACAC-3’; β-actin, forward: 5’- CATGTACGTTGCTATCCAGGC-3’ and reverse: 5’- CTCCTTA.

ATGTCACGCACGAT-3’.

### 2.13 Overexpression plasmid cloning and siRNA reagents

The following expression plasmids were generated: pEGFP-N1-ADAR, pcDNA3.1-flag-STUB1, pCMV-SPORT6-SYVN1, pCMV-SPORT6-TRIM25. The primers used in construction plasmids were designed by Sangon Biotech (Shanghai, China), pEGFP-N1-ADAR, forward: CGTCAGATCCGCTAGATGAATCCGCGGCAGGG and reverse: GTCCGGTAGCGCTAGTAC

TGGGCAGAGATAAAAGTTCTTTTCCT; pcDNA3.1-flag-STUB1, forward: TACCGAGCTCG

GATCATGAAGGGCAAGGAGGAGAAGG and reverse: CTCCGAATTCGGATCTGTAGTCC

TCCACCCAGCC; pCMV-SPORT6-SYVN1, forward: GGGAATTCCGGACCGATGTTCCGCA

CGGCAGT and reverse: CAAAAAAGCAGGCTGTCAGTGGGCAACAGGAGACTCC; pCMV-SPORT6-TRIM25, forward: GGGAATTCCGGACCGATGGCAGAGCTGTGCCC and reverse: CAAAAAAGCAGGCTGCTACTTGGGGGAGCAGATGGAGA. ADAR (catalogue no. SIGS0001624-1) and STUB1(catalogue no. SIGS0001238-1) siRNA and non-silence control siRNA (NC siRNA) were purchased from Guangzhou RiboBio Co., Ltd. (Guangzhou, Guangdong, China).

### 2.14 Transwell assay

Transwell experiments were performed using the Corning Transwell kit. Firstly, T98G and U251 cells with ADAR overexpression (1.5 × 10^5^ cells per well) or ADAR knockdown (4 × 10^5^ cells per well) following 24 hours of transfection were collected and placed in the upper chamber of a 24-well plate containing 200 μL of foetal bovine serum (FBS) free medium. Simultaneously, 800 μL of culture medium containing 20% FBS was added to the lower chamber. Following a 24-hour incubation period, non-migrated cells were removed using a cotton swab. The remaining cells were fixed with 4% formaldehyde and stained with crystal violet. The assessment and quantification of cell migration ability was subsequently conducted using the analysis tool in Adobe Photoshop 2020.

### 2.15 E3 ubiquitin ligase prediction

Prediction of ADAR-targeting E3 ligases was performed utilizing the UbiBrowser 1.0 online platform (http://ubibrowser.bio-it.cn/ubibrowser/). The query parameters were configured as follows: “Substrate” was selected as the Query Protein type, “H. sapiens” was designated as the species, and “ADAR” was entered as the Submit Protein identifier.

### 2.16 Western blot

Cells were cultivated in 60 mm dishes to approximately 80% confluence and subsequently lysed with ice-cold RIPA lysis buffer (P0013B, Beyotime) containing 1% protease inhibitor mix. The concentration of the resulting supernate was determined by BCA method. Following the loading of target proteins onto an SDS polyacrylamide gel, the proteins were transferred from the gel to a PVDF membrane. Subsequently, membranes were incubated overnight at 4 °C with ADAR (14175, Cell Signaling Technology), STUB1 (55430-1-AP, Proteintech), Ubiquitin K48 (381517, ZEN-BIOSCIENCE), GFP antibody (Utibody, UM3002), Flag antibody (201126-3A6, ZEN-BIOSCIENCE) and β-actin antibody (GB15003, Servicebio). Following three washes with TBST, the membrane was then subjected to an incubation with secondary antibodies (511203 and 550108, ZEN-BIOSCIENCE) for a period of 70 minutes at room temperature. The detection and analysis of protein bands was conducted through the utilisation of enhanced ECL chemiluminescence, while the acquisition of digital images was facilitated by a Bio-Rad apparatus.

### 2.17 Co-immunoprecipitation

The co-immunoprecipitation (Co-IP) process was then executed by employing protein A/G PLUS agarose beads (Santa Cruz Biotechnology, SC-2003). The subsequent procedure was as follows: samples were extracted from HEK 293T cells (10 cm Petri dishes were used for each sample) and lysed using immunoprecipitation lysate. Samples were then subjected to incubation with either a mouse GFP antibody (1:100, Utibody, UM3002) or a mouse isotype control IgG (1:150, Abclonal, AC005, China) at 4 °C for a duration of 16 hours, with gentle rotation. Subsequently, 30 μL of protein A/G beads were added to the samples and incubated overnight at 4 °C. The beads were subjected to a washing process comprising five cycles of cold IP lysis buffer. The eluted proteins were then utilised for the purpose of Western blotting.

### 2.18 Statistical analysis

All experiments were conducted in triplicate with independent replicates. The data are expressed as the mean ± standard error of the mean (SEM). Statistical comparisons between two groups were performed using Student’s t-test. For bioinformatic analyses, gene expression differences in unpaired samples were evaluated using Wilcoxon Rank Sum and Signed Rank Tests. The statistical analyses were implemented using R software (version 4.0.3) and GraphPad Prism 7.0. The specific statistical methodologies employed in the individual experiments are documented in the corresponding Materials and Methods sections or figure legends. Statistical significance was defined as *p* < 0.05.

## 3 Results

### 3.1 Analysis of ADAR expression in pan-cancer

**Figure 1:**
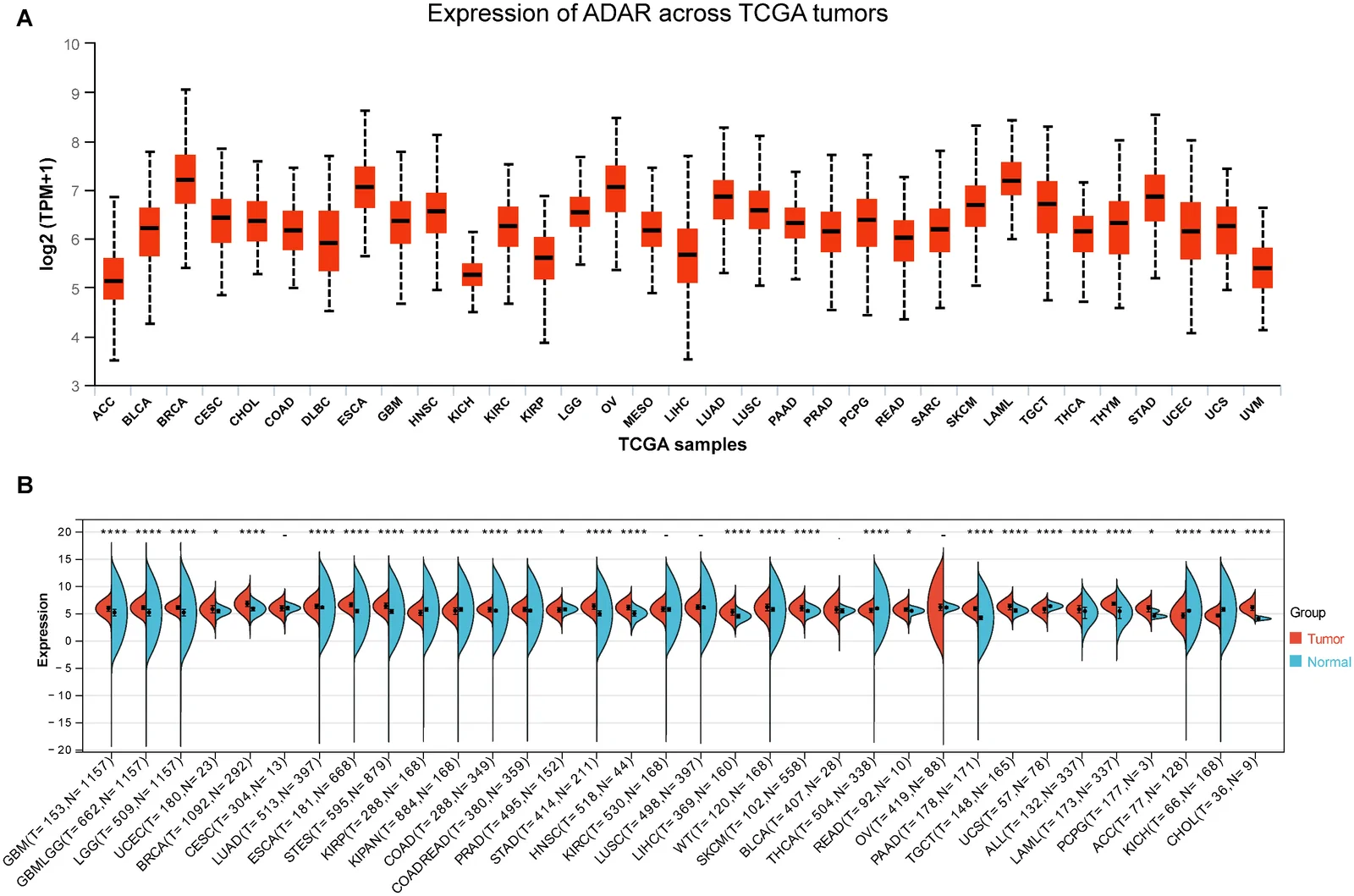
ADAR expression profiles across TCGA tumours and comparison with normal tissues. **(A)** Box plot depicting the expression levels of ADAR (log2[TPM + 1]) across 33 cancer types in TCGA dataset. Each box represents the interquartile range with the median indicated by the horizontal line. Whiskers extend to 1.5 times the interquartile range. **(B)** Violin plots showing the distribution of ADAR expression in tumour (red) versus normal (blue) tissues across multiple cancer types. The width of each violin indicates the density of data points at the corresponding expression level. Sample sizes are indicated for each cancer type (N=number of samples). Asterisks above the plots denote statistical significance of expression differences between tumour and normal tissues (*p < 0.05, **p < 0.01, ***p < 0.001, ****p < 0.0001).

Current studies on ADAR expression in multiple cancer types are more limited. In order to provide a more comprehensive assessment of ADAR expression in cancer, a study was conducted of ADAR mRNA expression in pan-cancer. A comprehensive analysis of ADAR expression across diverse cancer types in the TCGA dataset has revealed significant heterogeneity in expression patterns. As demonstrated in **Figure 1A**, the levels of ADAR expression (measured as log2[TPM + 1]) exhibited significant variation among diverse cancer types, with the highest median expression observed in breast invasive carcinoma (BRCA), skin cutaneous melanoma (SKCM), and thyroid carcinoma (THCA). In contrast, adrenocortical carcinoma (ACC), uveal melanoma (UVM), and mesothelioma (MESO) exhibited comparatively reduced ADAR expression.

A comparison of ADAR expression between tumour and corresponding normal tissues (see **Figure 1B**) revealed a significant differential expression pattern across multiple cancer types. The violin plots demonstrate that ADAR is predominantly dysregulated in most cancer types, with significant upregulation (denoted by asterisks) observed in numerous cancers including glioblastoma multiforme (GBM), breast invasive carcinoma (BRCA), and kidney renal clear cell carcinoma (KIRC). The consistent pattern of differential expression across diverse cancer types suggests a potential role for ADAR in tumourigenesis across multiple tissues of origin.

### 3.2 Prognostic analysis of ADAR

**Figure 2:**
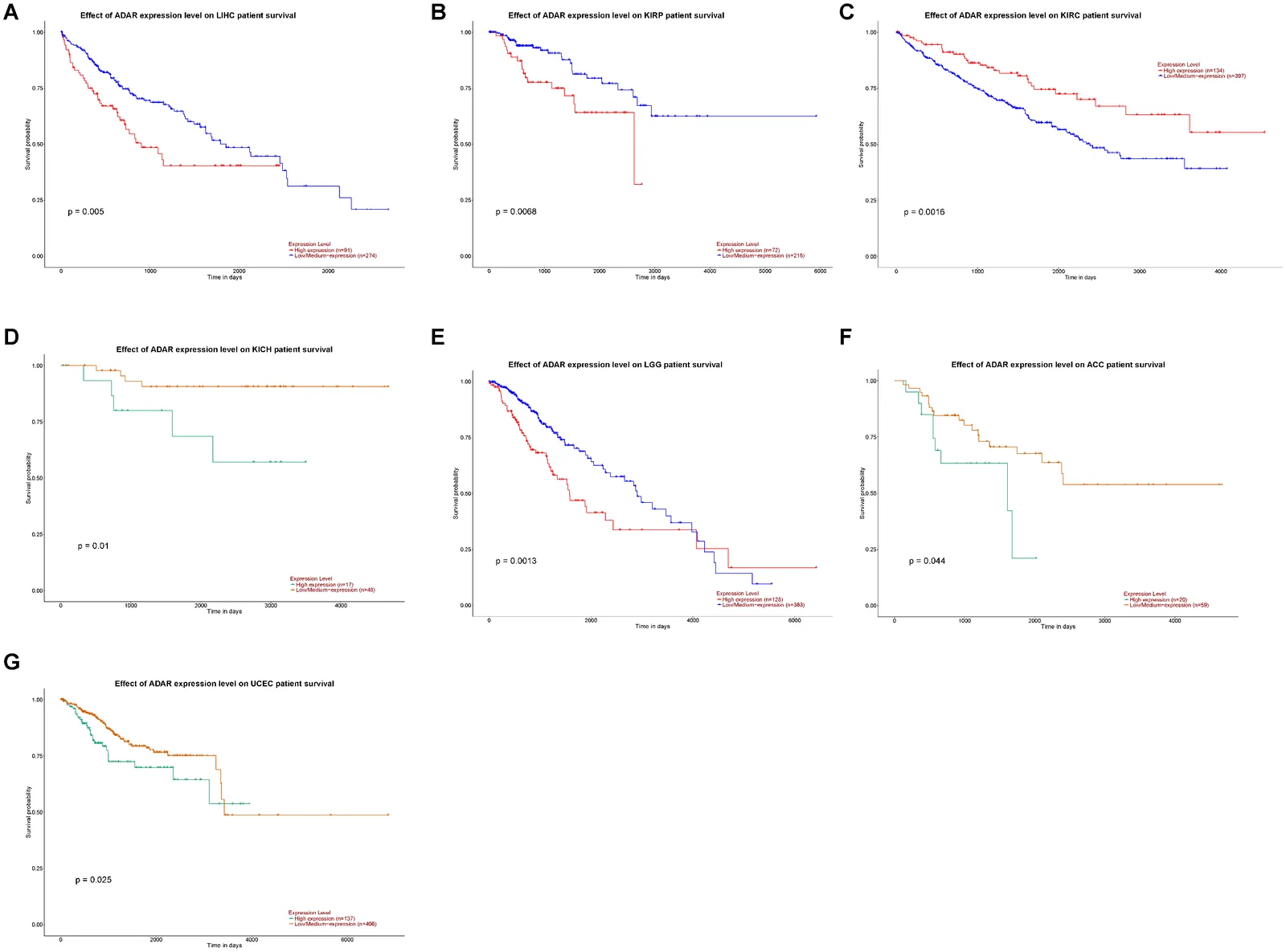
Impact of ADAR expression on patient survival across multiple cancer types from TCGA. Kaplan-Meier survival curves showing the relationship between ADAR expression levels and overall survival in **(A)** liver hepatocellular carcinoma (LIHC), **(B)** kidney renal papillary cell carcinoma (KIRP), **(C)** kidney renal clear cell carcinoma (KIRC), **(D)** kidney chromophobe (KICH), **(E)** brain lower grade glioma (LGG), **(F)** adrenocortical carcinoma (ACC), and **(G)** uterine corpus endometrial carcinoma (UCEC). Patients were stratified into high expression (red lines) and low expression (blue or green lines) groups based on median ADAR expression levels. *P*-values were calculated using the log-rank test. The number of patients in each group is indicated in the legend of each panel. Time is represented in days on the x-axis, and survival probability on the y-axis.

We evaluated the prognostic significance of ADAR expression in multiple cancer types in the TCGA dataset by Kaplan-Meier survival analysis using the UALCAN database. As shown in **Figure 2**, we listed the cancer types with statistically significant prognosis. The results showed that there was a significant correlation between ADAR expression levels and patient survival outcomes, but the direction of the effect differed significantly among cancer types.

In the context of liver hepatocellular carcinoma (LIHC; **Figure 2A**), kidney renal papillary cell carcinoma (KIRP; **Figure 2B**), and brain lower grade glioma (LGG; **Figure 2E**), patients exhibiting high ADAR expression demonstrated significantly poorer survival outcomes in comparison to those exhibiting low expression (p = 0.005, p = 0.0068, and p = 0.0013, respectively). Conversely, for kidney renal clear cell carcinoma (KIRC; **Figure 2C**), kidney chromophobe (KICH; **Figure 2D**), adrenocortical carcinoma (ACC; **Figure 2F**), and uterine corpus endometrial carcinoma (UCEC; **Figure 2G**), elevated ADAR expression was correlated with markedly enhanced survival outcomes (p = 0.0016, p = 0.01, p = 0.044, and p = 0.025, respectively).

These findings underscore the intricate and context-dependent function of ADAR in cancer progression. The divergent associations between ADAR expression and patient prognosis across different cancer types suggest that ADAR may function as either a tumour suppressor or an oncogene depending on the tissue-specific molecular context, potentially reflecting differences in RNA editing requirements or interactions with tissue-specific factors in various cancer types.

### 3.3 Analysis of ADAR gene mutation and expression level

**Figure 3:**
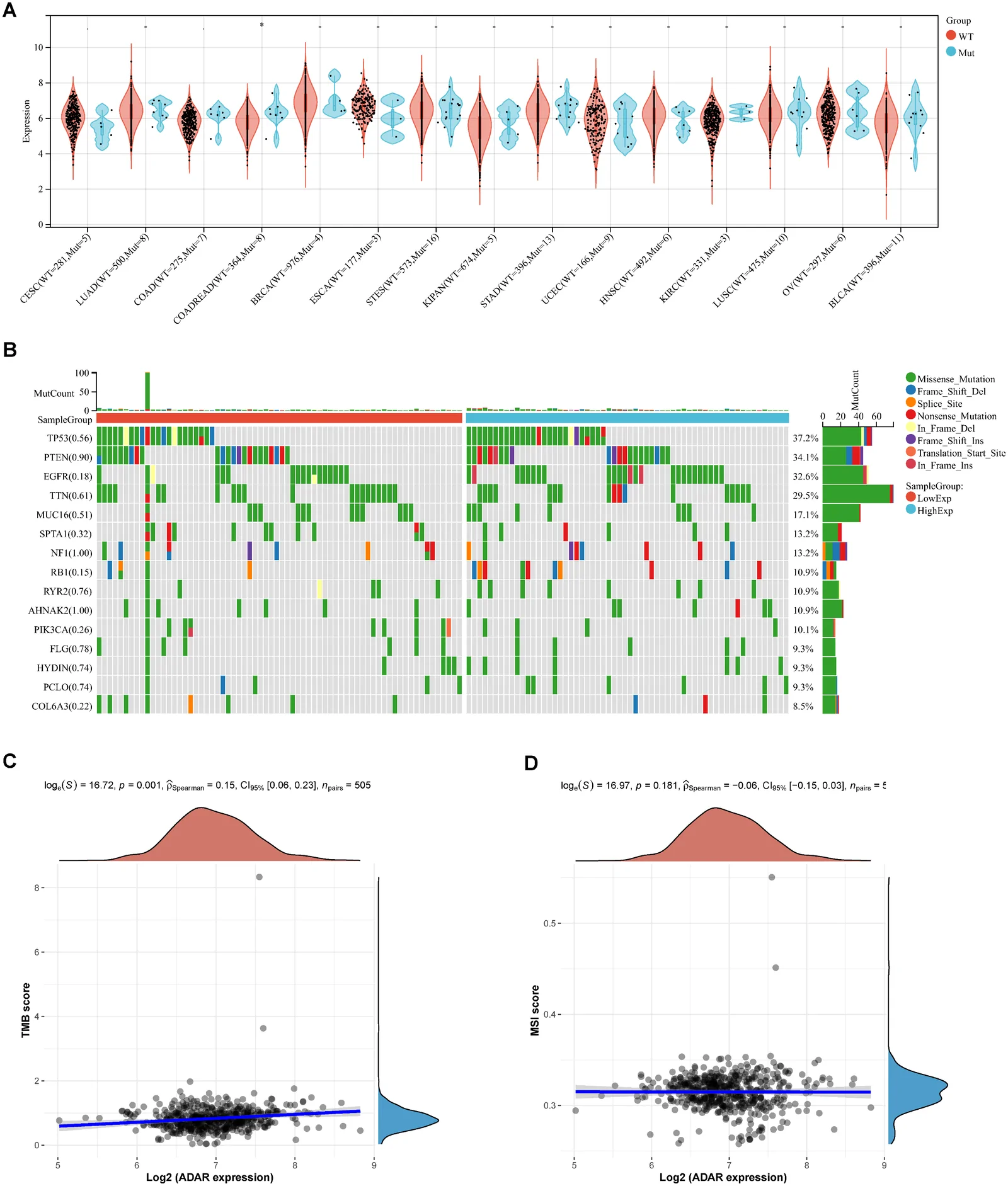
Association between ADAR expression, mutation status across cancer types, and genomic instability in glioblastoma multiforme. **(A)** Violin plots showing the distribution of ADAR expression in wild-type (WT, red) versus mutated (mut, blue) samples across 16 cancer types. Each dot represents an individual sample, with sample numbers indicated for each cancer type. **(B)** Oncoplot displaying mutation profiles of the top 15 most frequently mutated genes in GBM samples stratified by ADAR expression (high expression: red bar; low expression: blue bar). Different mutation types are color-coded according to the legend. Numbers and percentages on the right indicate mutation frequency of each gene. The upper bar chart shows mutation count per sample. **(C)** Correlation between ADAR expression (log2) and TMB score in GBM. The scatter plot shows a significant positive correlation (*p* = 0.001, Spearman’s ρ = 0.15). **(D)** Correlation between ADAR expression (log2) and MSI score in GBM, showing no significant association (*p* = 0.181, Spearman’s ρ = -0.06). In panels C and D, the blue line represents the linear regression fit, with density plots shown on the top and right margins.

A comprehensive analysis of ADAR expression in relation to mutation status across multiple cancer types from TCGA was conducted. Given the frequent association between Simple Nucleotide Variation (SNV) status and abnormal gene expression, the SNV status of ADAR was initially investigated. An exhaustive investigation was undertaken utilising the TCGA Pan-Cancer data set, which yielded the discovery that ADAR exhibited mutations in 16 distinct cancer types. The violin plots provide a visual representation of the disparity in ADAR expression patterns between wild-type (WT) and mutated (Mut) samples across these cancer types, including CESC (WT = 281, Mut = 5), LUAD (WT = 500, Mut = 8), and COAD (WT = 275, The following mutations were identified: COADREAD (WT = 364, Mut = 8), BRCA (WT = 976, Mut = 4), ESCA (WT = 177, Mut = 3), STES (WT = 573, Mut = 16), KIPAN (WT = 674, Mut = 5), and STAD (WT = 396, Mut = 13). The UCEC (WT = 166, Mut = 9), HNSC (WT = 492, Mut = 6), KIRC (WT = 331, Mut = 3), LUSC (WT = 475, Mut = 10), OV (WT = 297, Mut = 6), and BLCA (WT = 396, Mut = 11) (**Figure 3A**) were found to be significantly associated with the presence of the mutation.

Given the particularly significant association between ADAR expression and patient survival in brain lower grade glioma (LGG) observed in our previous survival analysis (**Figure 2E**), we further investigated the relationship between ADAR and genomic alterations specifically in glioblastoma multiforme (GBM). We stratified GBM samples into high and low ADAR expression groups and examined mutation profiles of key cancer-associated genes (**Figure 3B**), with the differentially mutated genes documented in **Supplementary Table 3**. The oncoplot reveals distinct mutational patterns between these groups, with notable differences in mutation frequencies of critical tumour suppressor genes and oncogenes. TP53 showed the highest mutation frequency (37.2%), followed by PTEN (34.1%), EGFR (32.6%), and TTN (29.5%). Different mutation types are represented by distinct colours, with missense mutations (green) being the predominant type. Samples with high ADAR expression (red bar) displayed different mutation patterns compared to those with low expression (blue bar).

We further investigated whether ADAR expression correlates with genomic instability in GBM using two established metrics: tumour mutation burden (TMB) and microsatellite instability (MSI). The correlation analysis revealed a significant positive association between ADAR expression and TMB (**Figure 3C**; *p* = 0.001, Spearman’s *ρ* = 0.15, 95% CI [0.06, 0.23]), suggesting that higher ADAR expression is associated with increased mutation load in GBM. In contrast, no significant correlation was observed between ADAR expression and MSI scores (**Figure 3D**; *p* = 0.181, Spearman’s *ρ* = -0.06, 95% CI [-0.15, 0.03]).

These findings suggest that ADAR may play a role in modulating genomic stability in GBM, potentially through its RNA editing function, which could influence DNA damage response pathways or mutation repair mechanisms.

### 3.4 ADAR correlates with oncogenic pathways in GBM

To further characterize the functional implications of ADAR expression in glioblastoma multiforme (GBM), we performed correlation analyses between ADAR expression levels and various oncogenic pathway activities and tumour microenvironment features.

**Figure 4:**
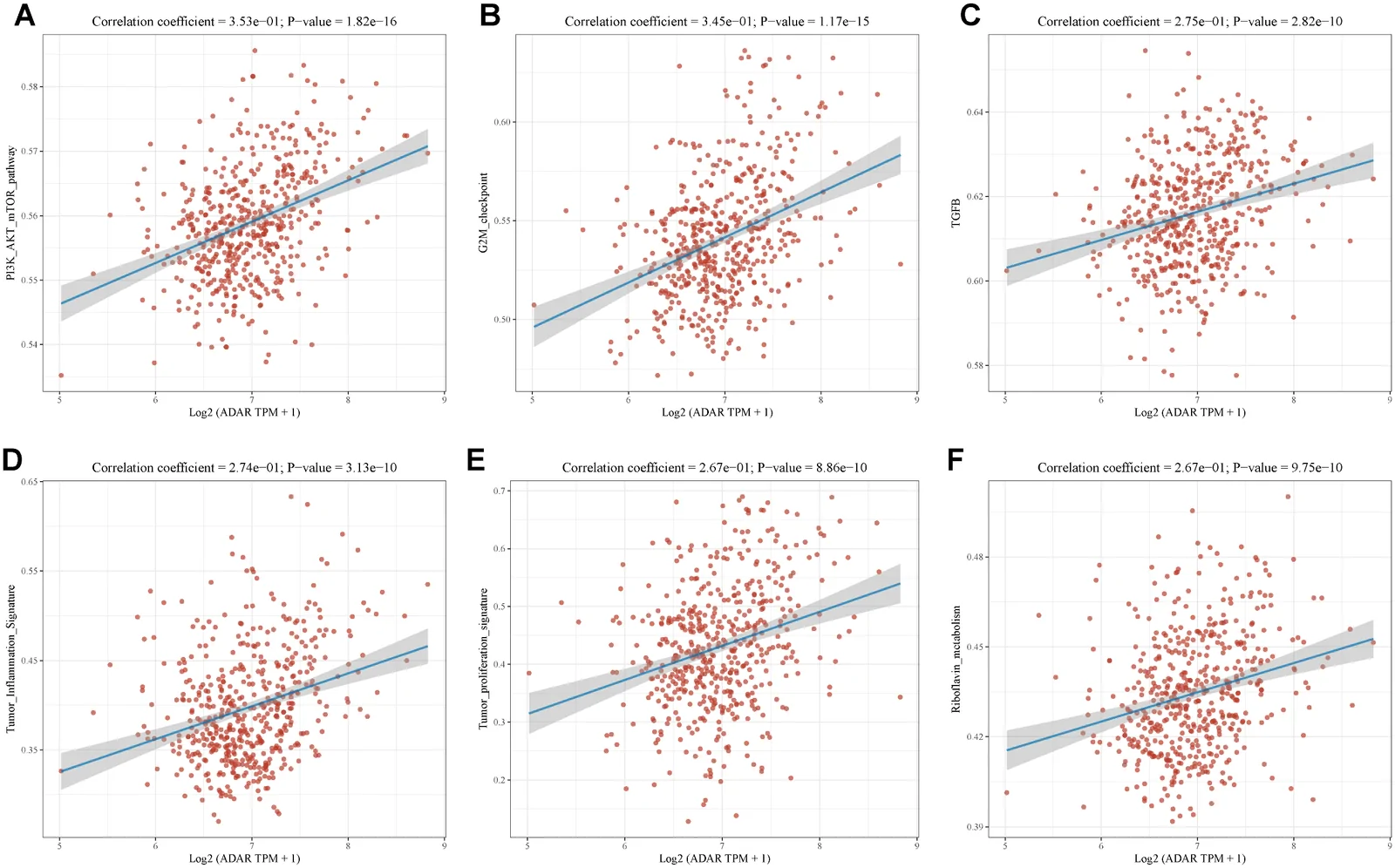
Correlation between ADAR expression and oncogenic pathways and tumour microenvironment features in glioblastoma multiforme. Scatter plots showing the correlation between ADAR expression (Log2(ADAR TPM + 1)) and various pathway activity scores: **(A)** PI3K-AKT-mTOR pathway, **(B)** G2M checkpoint pathway, **(C)** TGFβ signalling, **(D)** Tumour inflammatory signature, **(E)** Tumour proliferation signature, and **(F)** Ribosomal metabolism. Each dot represents an individual low-grade glioma sample. The blue line indicates the linear regression fit with 95% confidence interval shown in gray. Correlation coefficients and p-values are displayed at the top of each panel. All correlations were statistically significant (*p* < 0.001), with correlation coefficients ranging from 0.267 to 0.353.

Our analyses of low-grade glioma samples revealed significant positive correlations between ADAR expression and key cancer-related signalling pathways. As shown in **Figure 4**, ADAR expression (measured as log2(ADAR TPM + 1)) exhibited a strong positive correlation with PI3K-AKT-mTOR pathway activity (correlation coefficient = 0.353, *p* = 1.82e-16; **Figure 4A**). Similarly, G2M checkpoint pathway activity showed a significant positive association with ADAR expression (correlation coefficient = 0.345, *p* = 1.17e-15; **Figure 4B**). ADAR expression also demonstrated significant positive correlations with various immune and stromal signatures in the GBM microenvironment. TGFβ signalling, a critical regulator of tumour progression and immune suppression, positively correlated with ADAR expression (correlation coefficient = 0.275, *p* = 2.82e-10; **Figure 4C**). Tumour inflammatory signature showed a correlation coefficient of 0.274 (*p* = 3.13e-10; **Figure 4D**). Furthermore, we observed significant associations between ADAR expression and tumour proliferation signature (correlation coefficient = 0.267, *p* = 8.86e-10; **Figure 4E**) and ribosomal metabolism (correlation coefficient = 0.267, *p* = 9.75e-10; **Figure 4F**). The correlation between ADAR gene expression in low-grade glioma and all pathways is shown in Supplementary file 1.

These findings collectively suggest that ADAR may play a crucial role in modulating multiple oncogenic pathways and tumour microenvironment features in GBM. The consistent positive correlations with proliferation, cell cycle regulation, and immune-related pathways indicate that ADAR might function as an important regulator of GBM progression through its influence on multiple cancer hallmarks.

### 3.5 Expression distribution and cell type specificity of ADAR in a single-cell atlas of glioblastoma

Following our discovery that ADAR is positively correlated with multiple cancer-related signalling pathways in GBM, we further explored the expression characteristics of ADAR in the glioblastoma microenvironment. Using single-cell RNA sequencing data from the Cancer Single-cell Expression Map (https://ngdc.cncb.ac.cn/cancerscem/index) database, we conducted an in-depth analysis of the expression pattern of ADAR in GBM.

**Figure 5:**
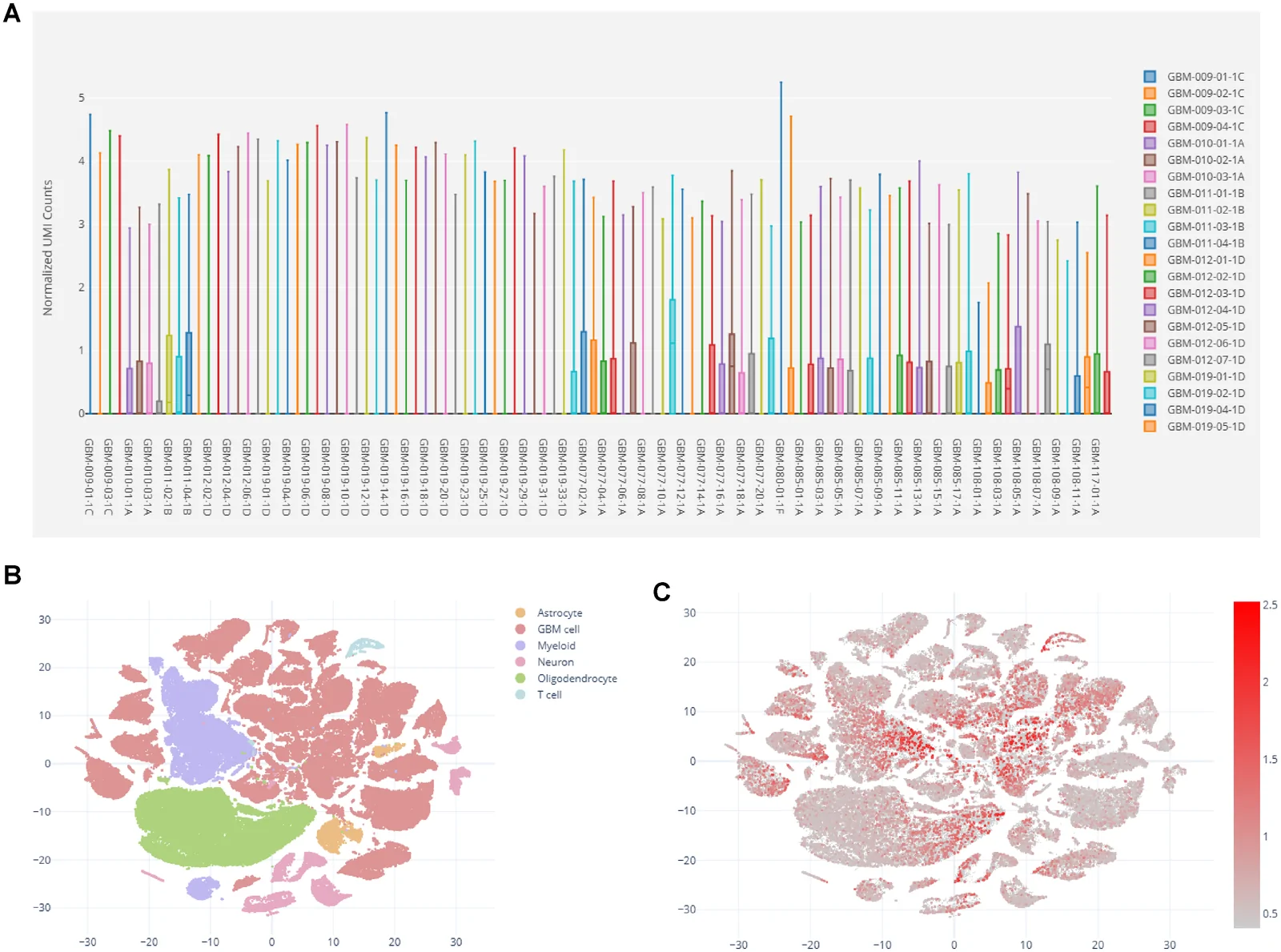
Single-cell RNA sequencing data reveal the expression pattern of ADAR in the glioblastoma microenvironment. **(A)** Normalised unique molecular identifier (UMI) counts of the ADAR gene in different GBM samples, showing the expression heterogeneity of ADAR across different patient samples. **(B)** UMAP dimensionality reduction clustering analysis of GBM single-cell transcriptomics data, showing the cell type composition in the tumour microenvironment, including GBM tumour cells (red), astrocytes (orange), oligodendrocytes (purple), neurons (pink), oligodendrocytes (green), and T cells (blue). **(C)** Heatmap of ADAR expression levels in the GBM tumour microenvironment. Colours ranging from grey to red indicate expression levels from low to high, showing that ADAR is highly expressed in GBM tumour cells and some myelinating cells.

As shown in **Figure 5A**, ADAR exhibited significant expression differences across different GBM patient samples, particularly in the GBM-009 series samples, where ADAR showed high expression levels, while expression levels were relatively low in the GBM-012 and GBM-019 series samples. This expression heterogeneity suggests that ADAR may play a differential role in different GBM subtypes.

To precisely localise ADAR expression in the GBM microenvironment, we performed UMAP dimensionality reduction clustering analysis on single-cell data (**Figure 5B**). The results revealed that the GBM microenvironment comprises multiple cell types, including dominant GBM tumour cells (red), infiltrating immune cells (e.g., oligodendrocytes and T cells), and brain tissue-resident cells (e.g., neurons and oligodendrocytes). Notably, ADAR expression exhibited significant differences across these cell types (**Figure 5C**). ADAR showed the highest expression levels in GBM tumour cells (red regions), followed by some medullary cells, while expression levels were relatively low in normal neurons and oligodendrocytes. This cell type-specific expression pattern strongly suggests that ADAR may primarily function in tumour cells and potentially influence the formation of the tumour immune microenvironment.

### 3.6 ADAR is upregulated in glioblastoma and promotes migration *in vitro*

**Figure 6:**
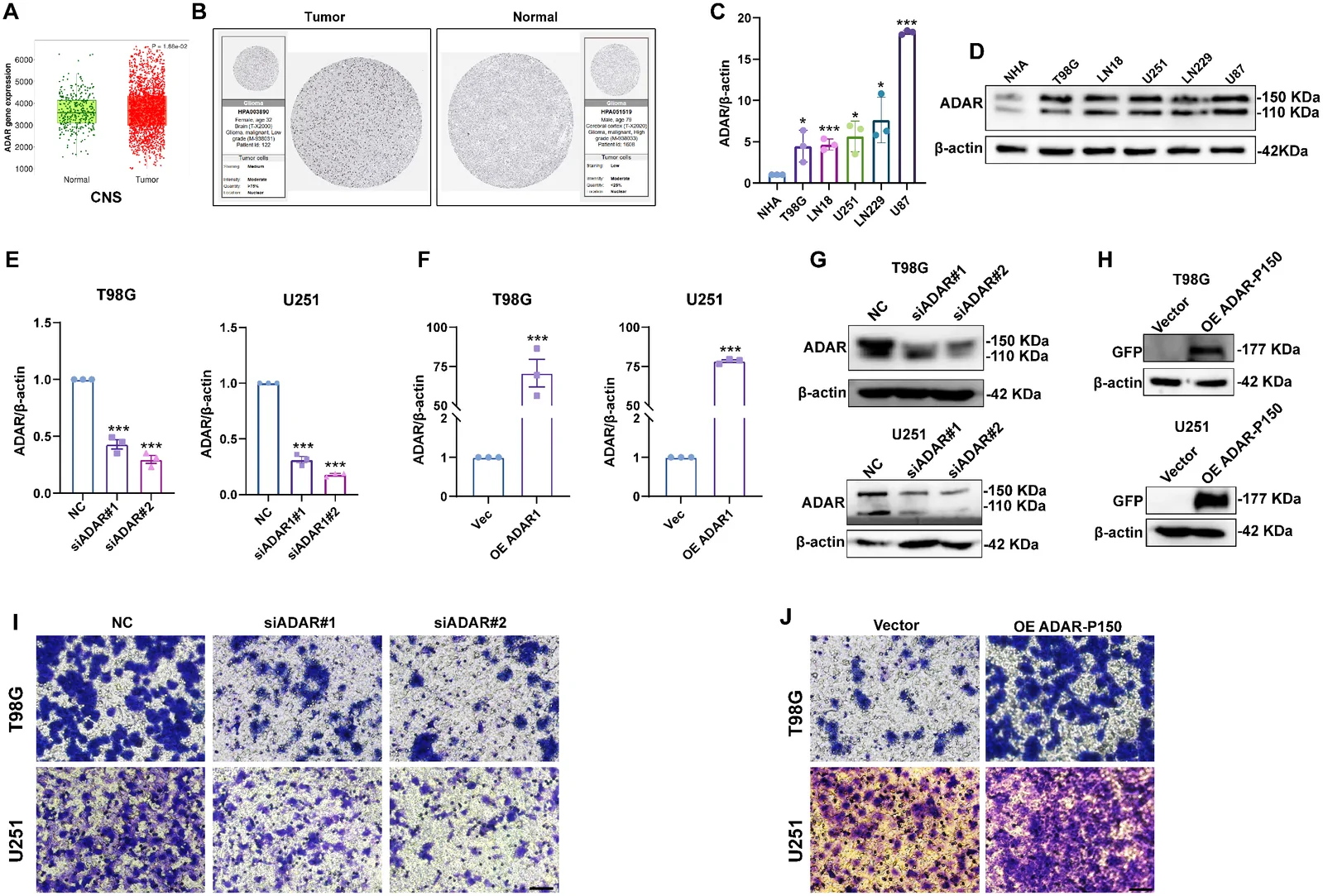
ADAR is upregulated and promotes cell migration in glioblastoma. **(A)** Expression levels of ADAR in CNS tumours (red) versus normal brain tissue (green) based on TCGA data in the UALCAN database. **(B)** Representative images of immunohistochemical staining of ADAR in human glioblastoma tissue and normal brain tissue. **(C)** qPCR analysis showing the expression levels of ADAR mRNA in normal human astrocytes (NHA) and five glioma cell lines (T98G, LN18, U251, LN229 and U87). Data are expressed as mean ± standard error (SE). *p < 0.05, ***p < 0.001 compared with NHA. **(D)** Western blot analysis of ADAR protein expression in NHA and five glioma cell lines. β-actin was used as an internal reference. **(E)** Validation of ADAR knockdown efficiency by two siRNAs in T98G and U251 cells by qPCR analysis. Data are expressed as mean ± standard error using β-actin as an internal reference. ***p < 0.001 compared to negative control (NC). **(F)** qPCR validation of ADAR overexpression in T98G and U251 cells. Data are expressed as mean ± standard error using β-actin as internal reference. ***p < 0.001 compared with vector control (Vector, Vec). **(G)** Western blot validation of ADAR knockdown in T98G and U251 cells. **(H)** Western blot validation of GFP-tagged ADAR-P150 overexpression in T98G and U251 cells. **(I)** Representative images of Transwell migration assay showing reduced invasion ability of T98G and U251 cells after ADAR knockdown compared to control. Scale bar = 100um. **(J)** Representative images of Transwell invasion assay showing enhanced invasion ability of T98G and U251 cells after ADAR-P150 overexpression compared to vector control. Scale bar = 100um.

Following our observation of high ADAR expression in glioblastoma cells in single-cell RNA sequencing data, we proceeded to validate its expression and functional roles experimentally. Analysis of data from the TCGA database revealed that ADAR expression was significantly elevated in central nervous system (CNS) tumour tissues compared to normal brain tissues (**Figure 6A**). This observation was further corroborated by immunohistochemistry staining of human GBM tissue, which demonstrated stronger ADAR staining in tumour tissues versus normal brain tissues (**Figure 6B**). To characterize ADAR expression across glioma cell lines, we performed qPCR analysis, which revealed that ADAR mRNA levels were markedly increased in various glioma cell lines (T98G, LN18, U251, LN229, and U87) compared to normal human astrocytes (NHA) (**Figure 6C**). Protein expression analysis by Western blot confirmed these findings, showing elevated ADAR protein levels across all tested glioma cell lines (**Figure 6D**). These results consistently demonstrate ADAR upregulation in GBM at both mRNA and protein levels. To investigate the functional significance of ADAR in GBM progression, we established both knockdown and overexpression systems in T98G and U251 cell lines. Using two different siRNAs targeting ADAR (siADAR#1 and siADAR#2), we achieved efficient knockdown of ADAR in both cell lines, as confirmed by qPCR and Western blot analyses (**Figures 6E, G**). Conversely, overexpression of ADAR-P150 isoform significantly increased ADAR levels in both cell lines (**Figures 6F, H**). With these cellular models, we next assessed the impact of ADAR on GBM cell migration using Transwell migration assays. ADAR knockdown dramatically reduced the invasive capacity of both T98G and U251 cells (**Figure 6I**). Consistently, overexpression of ADAR-P150 substantially enhanced the invasive potential of these cells (**Figure 6J**). These complementary loss- and gain-of-function experiments strongly suggest that ADAR plays a critical role in promoting GBM cell migration.

Collectively, these experimental findings align with our earlier bioinformatic analyses and provide direct evidence that ADAR is not only upregulated in GBM but also functionally important for driving migratory phenotypes. These results support the notion that ADAR may serve as a potential therapeutic target for controlling GBM progression.

### 3.7 Functional enrichment analysis of ADAR-associated genes in glioblastoma

To comprehensively understand the biological implications of ADAR dysregulation in GBM, we conducted a systematic functional enrichment analysis of ADAR-associated genes identified from the UALCAN database. Genes associated with ADAR in GBM are listed in **Supplementary Table 2**. This analysis provides a mechanistic framework to interpret the oncogenic functions of ADAR we observed in our experimental models.

**Figure 7:**
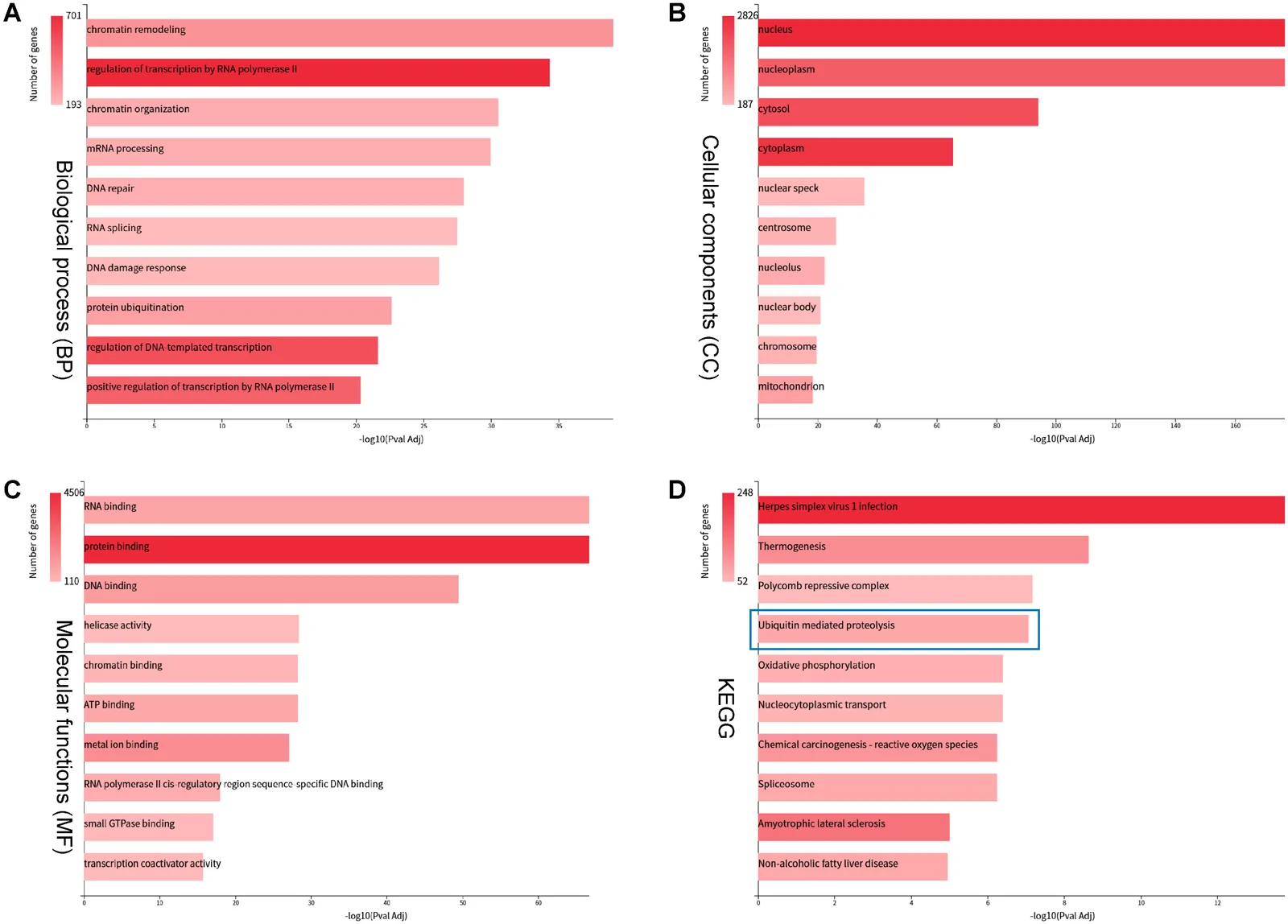
Functional enrichment analysis of ADAR-associated genes in glioblastoma. ADAR-associated genes were identified from the UALCAN database and subjected to functional enrichment analysis. **(A)** Top 10 enriched biological process (BP) terms. The x-axis represents -log10 (*P*-value adj), and the y-axis shows the enriched biological processes. The number of genes associated with each term is indicated on the left. **(B)** Top 10 enriched component (CC) terms, displaying the subcellular localization of ADAR-associated gene products. **(C)** Top 10 enriched molecular function (MF) terms, highlighting the predominant molecular activities of ADAR-associated genes. **(D)** Top 10 enriched KEGG pathways, revealing the signalling and metabolic pathways associated with ADAR-related genes in GBM. For all panels, bar length corresponds to statistical significance [-log10 (*P*-value adj)], and the number of genes in each category is indicated on the left side of each panel.

Gene Ontology (GO) analysis of biological processes (BP) revealed that ADAR-associated genes were significantly enriched in pathways related to chromatin remodelling, regulation of transcription by RNA polymerase II, and chromatin organization (**Figure 7A**). Additionally, these genes were involved in fundamental RNA processing pathways including mRNA processing, RNA splicing, and DNA damage response. The strong association with transcriptional regulation and RNA processing pathways aligns with ADAR’s canonical role in RNA editing and suggests broader effects on gene expression control in GBM. Cellular component (CC) analysis demonstrated that ADAR-associated genes were predominantly localized to the nucleus and nucleoplasm, with significant representation in the cytosol and cytoplasm (**Figure 7B**). This subcellular distribution is consistent with ADAR’s known nuclear localization and its involvement in both nuclear RNA processing and cytoplasmic functions in cancer cells. Analysis of molecular functions (MF) highlighted RNA binding and protein binding as the most significantly enriched functions among ADAR-associated genes (**Figure 7C**). DNA binding, helicase activity, and chromatin binding were also prominently featured, suggesting that ADAR may exert its effects through interactions with diverse molecular partners involved in nucleic acid metabolism and chromatin modification.

Kyoto Encyclopedia of Genes and Genomes (KEGG) pathway analysis identified herpes simplex virus 1 infection as the most significantly enriched pathway, followed by thermogenesis and polycomb repressive complex (**Figure 7D**). Notably, the significant enrichment of ubiquitin-mediated proteolysis pathway (highlighted in blue rectangles) suggested a potential regulatory relationship between the ubiquitin-proteasome system and ADAR. This finding was particularly interesting as protein degradation pathways often play critical roles in modulating the expression and function of key oncogenic proteins in cancer. Understanding the post-translational regulation of ADAR could provide critical insights into how its protein levels are maintained in cancer cells and potentially reveal new therapeutic vulnerabilities. Therefore, we will further investigate the ubiquitin-mediated proteasome degradation process of ADAR in glioblastoma cells and identify the specific molecular mechanisms involved in this process.

involved in this process.

### 3.8 STUB1 mediates the ubiquitin-proteasomal degradation of ADAR in glioblastoma

**Figure 8:**
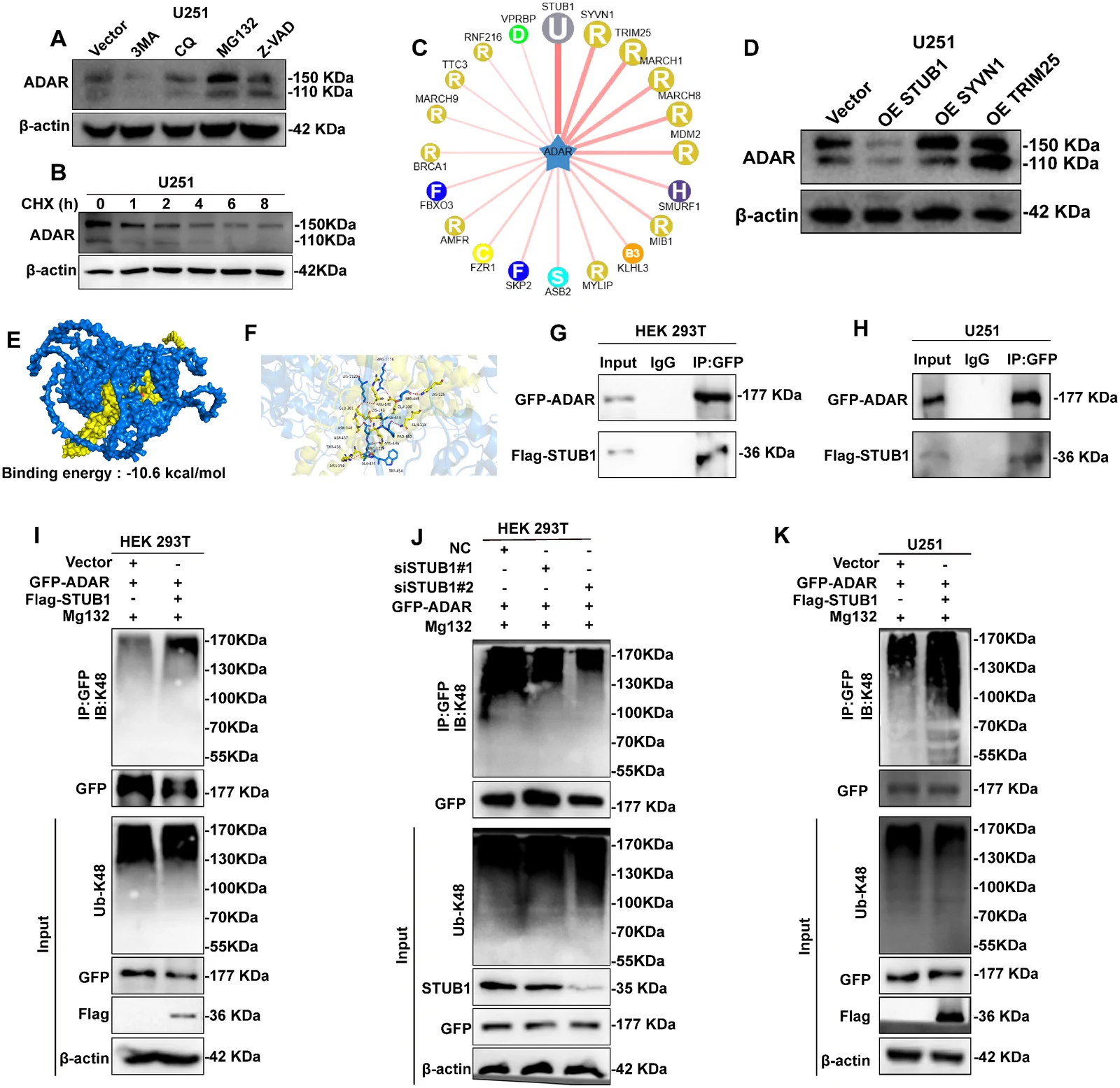
STUB1 mediates ubiquitin-proteasome degradation of ADAR. **(A)** Western blot analysis of ADAR protein levels in U251 cells treated with DMSO control, 3-MA, CQ, MG132, or Z-VAD. β-actin served as loading control. **(B)** Cycloheximide (CHX) chase assay in U251 cells. Cells were treated with CHX and harvested at the indicated time points (0, 1, 2, 4, 6, and 8 h). ADAR protein levels were detected by Western blot. β-actin served as loading control. **(C)** Network diagram from UbiBrowser database showing predicted E3 ubiquitin ligases for ADAR (blue node in centre). Node colours indicate E3 ligase families: yellow **(R)** for RING-type, green **(H)** for HECT-type, light blue **(U)** for U-box type, and orange **(O)** for other types. Edge thickness represents confidence level of predicted interactions. **(D)** Western blot analysis of endogenous ADAR levels in U251 cells overexpressing vector control, STUB1, SYVN1, or TRIM25. β-actin served as loading control. **(E)** Protein-protein docking model of ADAR (blue) and STUB1 (yellow) generated using GRAMM software, showing favourable binding energy of −10.6 kcal/mol. **(F)** Detailed view of the ADAR-STUB1 interaction interface highlighting key contact residues. **(G)** Co-IP analysis in HEK293T cells co-transfected with GFP-ADAR and Flag-STUB1. Cell lysates were immunoprecipitated with anti-GFP antibody or control IgG, followed by immunoblotting with indicated antibodies. **(H)** Co-IP analysis in U251 cells co-transfected with GFP-ADAR and Flag-STUB1. Cell lysates were immunoprecipitated with anti-GFP antibody or control IgG, followed by immunoblotting with indicated antibodies. **(I)** Ubiquitination assay in HEK293T cells transfected with GFP-ADAR alone or with Flag-STUB1, treated with MG132. GFP-ADAR was immunoprecipitated and ubiquitination detected with anti-K48-ubiquitin antibody. **(J)** Ubiquitination assay in HEK293T cells transfected with GFP-ADAR and control siRNA or STUB1 siRNAs, treated with MG132. GFP-ADAR was immunoprecipitated and ubiquitination detected with anti-K48-ubiquitin antibody. **(K)** Ubiquitination assay in U251 cells transfected with GFP-ADAR alone or with Flag-STUB1, treated with MG132. GFP-ADAR was immunoprecipitated and ubiquitination detected with anti-K48-ubiquitin antibody.

In light of our preceding discovery that the ubiquitin-proteasome pathway is substantially overrepresented in ADAR-associated genes, we embarked on a subsequent investigation to ascertain whether ADAR itself is subject to regulation by this pathway. Firstly, U251 cells were treated with a variety of inhibitors to probe the mechanism of ADAR degradation. Treatment with the proteasome inhibitor MG132 resulted in a significant accumulation of ADAR protein levels compared to control, whereas treatment with the autophagy inhibitors 3-Methyladenine (3MA) and chloroquine (CQ), as well as the pan-caspase inhibitor Z-VAD, showed minimal effects (**Figure 8A**), indicating that ADAR is primarily degraded through the ubiquitin-proteasome system rather than autophagy or caspase-mediated pathways. To further confirm the active turnover of ADAR protein, we performed a cycloheximide (CHX) chase assay in U251 cells. Following CHX treatment to block *de novo* protein synthesis, ADAR protein levels progressively decreased over time (**Figure 8B**), directly demonstrating that ADAR undergoes continuous protein turnover in GBM cells and further supporting its regulation at the post-translational level.

To identify specific E3 ubiquitin ligases that regulate ADAR degradation, we utilized the UbiBrowser database to predict potential E3 ligases for ADAR (**Figure 8C**). Among the candidates identified, the U-box domain-containing E3 ligase STUB1 (also known as CHIP) demonstrated the highest confidence score. To verify the functional relevance of these candidate E3 ligases, we overexpressed the top three candidates—STUB1, SYVN1, and TRIM25—in U251 cells. Notably, STUB1 overexpression significantly reduced endogenous ADAR protein levels, while SYVN1 and TRIM25 overexpression had no apparent effect (**Figure 8D**), indicating that STUB1 specifically promotes ADAR degradation in glioblastoma cells.

To further elucidate the structural basis of ADAR-STUB1 interaction, we performed protein-protein docking analysis using GRAMM software. The computational model revealed a favourable binding interface between ADAR (depicted in blue) and STUB1 (depicted in yellow), with a binding energy of -10.6 kcal/mol (**Figure 8E**). This notably negative binding energy suggests high affinity between these proteins. Detailed examination of the interaction interface (**Figure 8F**) revealed specific contact regions with several key amino acid residues positioned at the binding interface.

To confirm the direct interaction between ADAR and STUB1, we performed co-immunoprecipitation (Co-IP) experiments. In HEK293T cells co-expressing GFP-ADAR and Flag-STUB1, immunoprecipitation with anti-GFP antibody successfully pulled down Flag-STUB1, while control IgG showed no interaction (**Figure 8G**). Importantly, we also validated this interaction in glioblastoma cells by performing Co-IP in U251 cells, where STUB1 was detected in GFP-ADAR immunoprecipitates (**Figure 8H**), confirming that the STUB1-ADAR interaction occurs in the relevant cellular context.

Next, we investigated whether STUB1 promotes ADAR ubiquitination. In HEK293T cells treated with MG132, co-expression of Flag-STUB1 with GFP-ADAR significantly enhanced K48-linked polyubiquitination of ADAR compared to cells expressing GFP-ADAR alone (**Figure 8I**). K48-linked ubiquitination is a classical signal for proteasomal degradation. To further confirm STUB1’s role in ADAR ubiquitination, we performed the reciprocal experiment by knocking down STUB1. In HEK293T cells expressing GFP-ADAR, knockdown of STUB1 using two independent siRNAs markedly reduced K48-linked ubiquitination of ADAR compared to control siRNA (**Figure 8J**). Additionally, we validated STUB1’s effect on ADAR ubiquitination in glioblastoma cells by overexpressing Flag-STUB1 in U251 cells, which similarly enhanced ADAR ubiquitination (**Figure 8K**).

Collectively, these results demonstrate that STUB1 functions as a specific E3 ubiquitin ligase for ADAR, promoting its K48-linked polyubiquitination and subsequent proteasomal degradation. This newly identified STUB1-ADAR regulatory axis provides important insights into the post-translational control of ADAR protein levels in GBM cells and suggests that targeting this interaction may represent a novel therapeutic strategy for glioblastoma.

## 4 Discussion

Our study has established the crucial role of ADAR in the progression of glioblastoma and revealed a novel mechanism through STUB1-mediated ubiquitination regulation. Through multi-omics analysis and experimental validation, we found that ADAR expression levels vary across different cancer types, with significantly elevated expression in glioblastoma tumour cells. Functionally, ADAR promotes glioblastoma cell migration, and its expression is associated with key oncogenic pathways such as the PI3K-AKT-mTOR and G2M checkpoint pathways. Most importantly, we identified STUB1 as a specific E3 ubiquitin ligase that regulates ADAR protein stability through K48-linked ubiquitination, leading to its proteasome-mediated degradation.

These findings further consolidate the role of ADAR in cancer development as previously reported. While earlier studies emphasised the widespread role of ADAR in glioblastoma development (8, 17), this study expands this understanding by elucidating specific post-translational mechanisms regulating ADAR protein levels. Previous pan-cancer analyses primarily focused on ADAR’s transcriptional expression patterns and immune-related functions (3, 18), whereas this study delves into protein stability regulation, providing a more comprehensive picture of ADAR homeostasis in cancer.

The identification of STUB1 as an ADAR regulatory factor aligns with recent studies on the role of STUB1 in cancer biology (13, 19). While previous studies have confirmed the function of STUB1 as an E3 ubiquitin ligase in various pathological environments (12, 20), its specific interaction with ADAR in glioblastoma is a new finding. Our results suggest that the STUB1-ADAR axis constitutes a previously unrecognised regulatory mechanism with significant implications for GBM progression. Notably, the functional relevance of the STUB1-ADAR axis may be further shaped by the unique pathological features of the GBM microenvironment, particularly hypoxia. Glioblastomas are characterised by extensive hypoxic regions, with pseudopalisading necrosis representing hypoxia-driven tumour cell migration (21). STUB1/CHIP functions as a stress-responsive E3 ubiquitin ligase and co-chaperone whose activity is dynamically regulated by the availability of molecular chaperones such as Hsp70 and Hsp90 (22, 23). Given that hypoxia profoundly alters chaperone expression and the proteostasis network, it is plausible that hypoxic conditions modulate STUB1-mediated ubiquitination and degradation of ADAR. Furthermore, RNA editing dysregulation has been documented in GBM, with reduced editing of substrates such as GluR-B and miR-376a* contributing to tumour invasiveness (24, 25). Notably, ADAR1 has been implicated in HIF-1α stabilisation through editing-dependent mechanisms (26), suggesting potential crosstalk between hypoxia signalling and the RNA editing machinery. We speculate that under hypoxic stress, alterations in STUB1 activity or chaperone availability may lead to dysregulated ADAR protein turnover, consequently reshaping the RNA editome and promoting GBM progression and therapeutic resistance.

Notably, our single-cell analysis also revealed high ADAR expression in myeloid cells within the GBM microenvironment. This is consistent with ADAR1’s well-known role in innate immune regulation, where its interferon-inducible p150 isoform edits endogenous double-stranded RNA to prevent aberrant MDA5-mediated immune activation (27, 28). Tumour-associated macrophages and microglia in GBM are chronically exposed to inflammatory signals, likely sustaining elevated ADAR expression. This raises the possibility that ADAR may also contribute to GBM progression through a non-cell-autonomous mechanism by modulating the immunosuppressive microenvironment, which warrants further investigation with cell type-specific approaches.

However, this study has several limitations that require further investigation. First, our functional experiments were conducted *in vitro* and may not fully replicate the complex tumour microenvironment of GBM. Second, although we established the role of STUB1 in regulating ADAR protein levels, we have not yet fully characterised the specific ubiquitinylation sites on ADAR or the structural determinants of the STUB1-ADAR interaction. Third, while we confirmed the impact of ADAR on cell migration, we did not explore all potential downstream targets of ADAR-mediated RNA editing that may contribute to this phenotype in GBM.

To overcome these limitations, future studies should incorporate *in vivo* models to validate the role of the STUB1-ADAR axis in a physiologically relevant context. Mass spectrometry can identify specific ubiquitinylation sites on ADAR, while structural biology techniques can reveal the molecular interaction interface between STUB1 and ADAR. Additionally, RNA sequencing of ADAR-regulated cells can help identify specific RNA editing events that promote GBM progression.

Looking ahead, our findings raise several thought-provoking questions. First, how does the STUB1-ADAR regulatory axis respond to common cellular stresses in the tumour microenvironment, such as hypoxia or nutrient deprivation? Second, can the STUB1-ADAR interaction serve as a therapeutic target, such as through small-molecule inhibitors or peptide-based approaches? Third, does this regulatory mechanism exist in other cancer types with high ADAR expression, and if so, are there tissue-specific regulators of this interaction? Fourth, how does ADAR regulation interact with other post-translational modifications to form a comprehensive regulatory network governing RNA editing in cancer?

Additionally, the role of ADAR in regulating immune therapy responses warrants further investigation, particularly given the emerging importance of RNA editing in shaping the tumour immune microenvironment. The significant correlation we observed between ADAR expression and tumour mutation burden suggests its potential impact on the efficacy of immune checkpoint inhibitor therapy in glioblastoma (GBM), a cancer type renowned for its resistance to immune therapy.

In summary, the STUB1-ADAR regulatory axis we identified not only deepens our understanding of the molecular mechanisms of RNA editing in glioblastoma but also opens new avenues for therapeutic interventions. By targeting this specific protein interaction, it may be possible to regulate ADAR levels and activity, thereby providing a complementary therapeutic strategy for glioblastoma treatment.

## 5 Conclusions

This study identifies a novel STUB1-ADAR regulatory axis in glioblastoma that controls tumour cell migration. We demonstrated that ADAR is significantly upregulated in GBM and correlates with oncogenic pathways and poor survival outcomes. Through biochemical analysis, we established that E3 ubiquitin ligase STUB1 specifically mediates ADAR’s proteasomal degradation via K48-linked ubiquitination. This regulatory mechanism offers new insights into RNA editing control in cancer and presents a potential therapeutic target for GBM treatment. The STUB1-ADAR axis represents an important advance in understanding post-translational regulation in glioblastoma and may lead to novel approaches for managing this aggressive malignancy.

## Data Availability

The raw data supporting the conclusions of this article will be made available by the authors, without undue reservation.
